# Treating the host response: an alternative way to manage Ebola in Africa and the next influenza pandemic

**DOI:** 10.7189/jogh-09-010322

**Published:** 2019-06

**Authors:** David S Fedson

**Affiliations:** Retired physician, Sergy Haut, France

During the Ebola outbreak in West Africa in 2014-2015, more than 11 000 people died. More than 1000 people have dies in the re-emergent Ebola outbreak (May 2019) in the Democratic Republic of Congo (DRC). In both outbreaks, no licensed treatment has been available and in most treatment units, 50%-60% of patients have died. Many of them could probably have survived.

Patients who die of Ebola have elevated plasma levels of pro-inflammatory cytokines and widespread abnormalities of innate and adaptive immunity. These findings are associated with endothelial dysfunction and the loss of vascular barrier integrity [1-3]. Healthcare workers who were infected with Ebola virus in West Africa and evacuated for medical care were shown to have massive fluid losses. These losses were due to a dramatic increase in vascular permeability, a direct effect of endothelial dysfunction.

Several generic drugs, among them statins and angiotensin receptor blockers (ARBs), stabilize or restore vascular barrier integrity [1-3]. These drugs are safe when given to patients with acute critical illness and clinical studies suggest they might improve survival in patients with sepsis, pneumonia and influenza. In an article published online on August 25, 2014, this idea was proposed for treating Ebola patients [4]. Unfortunately, it was rejected by Ebola scientists and WHO staff [3] and it received no support from national health agencies or major foundations. Nonetheless, in November 2014 local physicians in Sierra Leone treated consecutively approximately 100 Ebola patients with a combination of atorvastatin (40 mg orally/day) and irbesartan (150 mg orally/day) [1-3]. Only three inadequately treated patients are known to have died.

There was no financial or logistical support to conduct a proper clinical trial; treatment was supported solely by a modest private donation. Sadly, local health officials refused to release information on this treatment experience. However, the many letters and memoranda they exchanged provided good evidence that treatment led to “remarkable improvement” in these patients [1-3].

Investigational Ebola treatments that were tested in West Africa (antiviral drugs, convalescent plasma, monoclonal antibodies) met with little success [5]. Unlike these treatments, atorvastatin and irbesartan primarily target the host response to the infection, not the virus [1-4]. In Sierra Leone, these drugs apparently stabilized endothelial function and restored normal fluid balance, helping Ebola patients live long enough to overcome their infection and survive. Unfortunately, investigational treatments that target the Ebola virus are being tested once again in the DRC. As before, host response treatment is being ignored.

Physicians are familiar with atorvastatin and irbesartan because most of them have used these drugs to treat patients with cardiovascular diseases. The drugs are produced as inexpensive generics and are widely available in Africa; a 10-day course of combination treatment would cost only a few dollars [3]. Details on the patients who were treated in Sierra Leone could have been externally reviewed and validated, but Ebola scientists and WHO staff have never shown any interest in doing so. Perhaps this is because targeting the host response instead of the virus is a new idea and they have many reasons not to accept it [3,6].

Generic drug combinations could include drugs other than statins and ARBs. They might be used to treat patients with other emerging virus diseases, including pandemic influenza [7-10]. These drugs could be critically important during the first six months, when it is estimated that almost 33 million people could die worldwide and no one will have access to pandemic vaccines. Generic drug combinations might also be used in the syndromic treatment of patients with everyday acute critical illnesses like seasonal influenza, sepsis and pneumonia [3,9,10]. In all of these conditions, an inability to overcome endothelial dysfunction (and other host abnormalities such as a failure of early mitochondrial biogenesis) can lead to multi-organ failure and death. If shown to be effective, these drugs would be available to everyone with access to basic health care. For pandemic influenza, combination treatment could be given in all countries on the first pandemic day.

**Figure:**
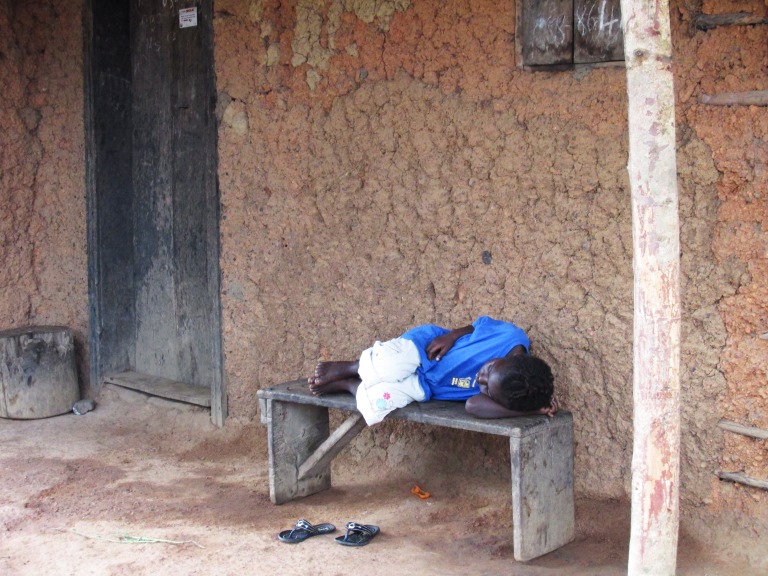
Photo: Entitled, “Waiting for an Ambulance”, this image was captured by CDC Public Health Advisor, Daniel W. Martin, MSPH. Too weak to sit up, this young girl in Masokory, Tonkolili District, Sierra Leone, was waiting for an ambulance to take her to the Ebola Holding Center in Magburaka, two and one half hours away (US Centers for Disease Control and Prevention). This image is in the public domain and thus free of any copyright restrictions.

Public health officials have a compelling reason to ensure that physicians undertake the clinical studies needed to show whether host response treatment with generic drugs will work [9,10]. This should be a central element of pandemic preparedness in all countries. To not undertake these studies will represent a colossal failure of both scientific and political imagination. For the next pandemic, the consequences of such failure could be unimaginable.
